# Combined Treatment with Immunotherapy-Based Strategies for MSS Metastatic Colorectal Cancer

**DOI:** 10.3390/cancers13246311

**Published:** 2021-12-16

**Authors:** Iosune Baraibar, Oriol Mirallas, Nadia Saoudi, Javier Ros, Francesc Salvà, Josep Tabernero, Elena Élez

**Affiliations:** 1 Department of Medical Oncology, Vall d’Hebron University Hospital, Passeig de la Vall d’Hebron, 119, 08035 Barcelona, Spain; omirallas@vhebron.net (O.M.); nsaoudi@vhio.net (N.S.); fjros@vhio.net (J.R.); fsalva@vhio.net (F.S.); jtabernero@vhio.net (J.T.); meelez@vhio.net (E.É.); 2 Vall d’Hebron Institute of Oncology (VHIO), 08035 Barcelona, Spain

**Keywords:** colorectal cancer, microsatellite stable, mismatch repair-proficiency, immunotherapy, immune checkpoint inhibitors, cold tumor, combined treatment

## Abstract

**Simple Summary:**

Given that most patients with MSS mCRC do not respond to immunotherapy agents or do not have durable clinical responses, there is an unmet clinical need to obtain predictors of response to immunotherapy and rational immunotherapy-based combination therapies for this entity. In this review, we present the efforts that have been made to date in the clinical setting to develop immunotherapy-based combinations for MSS mCRC and the rationale that lies behind each strategy.

**Abstract:**

In recent years, deepening knowledge of the complex interactions between the immune system and cancer cells has led to the advent of effective immunotherapies that have revolutionized the therapeutic paradigm of several cancer types. However, colorectal cancer (CRC) is one of the tumor types in which immunotherapy has proven less effective. While there is solid clinical evidence for the therapeutic role of immune checkpoint inhibitors in mismatch repair-deficient (dMMR) and in highly microsatellite instable (MSI-H) metastatic CRC (mCRC), blockade of CTLA-4 or PD-L1/PD-1 as monotherapy has not conferred any major clinical benefit to patients with MMR-proficient (pMMR) or microsatellite stable (MSS) mCRC, reflecting 95% of the CRC population. There thus remains a high unmet medical need for the development of novel immunotherapy approaches for the vast majority of patients with pMMR or MSS/MSI-low (MSI-L) mCRC. Defining the molecular mechanisms for immunogenicity in mCRC and mediating immune resistance in MSS mCRC is needed to develop predictive biomarkers and effective therapeutic combination strategies. Here we review available clinical data from combinatorial therapeutic approaches using immunotherapy-based strategies for MSS mCRC.

## 1. Introduction

Colorectal cancer (CRC) is the third most common primary tumor worldwide and ranks second in terms of mortality [1]. CRC is a heterogeneous disease that remains a largely unsolved clinical challenge. This heterogeneity characterizes CRC at several levels—including genomic, epigenomic, transcriptomic, and microenvironmental features [2]. Current management of metastatic CRC (mCRC) is based on the tumor molecular profile and the individual patient’s clinical condition. Validated biomarker-drug matches that guide treatment decisions in the metastatic setting are available, including *RAS/BRAF* mutations conferring resistance to anti-EGFR agents and *BRAF*-V600E mutations sensitizing tumors to anti-BRAF therapies. There is growing evidence for other biomarkers, including *HER2* amplifications.

Microsatellite instability (MSI) status is currently the main biomarker for immunotherapeutic response in mCRC. The advent of immunotherapy—including programmed cell death 1 (PD-1), programmed cell death-ligand 1 (PD-L1), and cytotoxic T-lymphocyte antigen 4 (CTLA-4) immune checkpoint inhibitors—has revolutionized the therapeutic paradigm across a wide range of cancers. It has increased treatment options, leading to remarkable improvements in terms of response rate, survival, and quality of life in many different tumor types, and has been positioned as frontline therapy, second-line therapy, and beyond in many solid tumors. The underlying mechanism of action relies on the upregulation of the immune system to reverse the immunosuppression provoked by cancer cells to evade an antitumor T cell response. In CRC, the beneficial effect of immune checkpoint inhibitors observed in patients with mCRC harboring deficient mismatch repair (dMMR) and MSI has been ascribed to the abundance of DNA replication errors due to the loss of function of any of the DNA mismatch machinery proteins, including MSH2, MSH6, MLH1, and PMS2 [3], that leads to a hypermutated phenotype. These tumors are characterized by high tumor mutational burden (TMB) and by consequence an inflammatory tumor microenvironment (TME), comprising infiltrating lymphocytes (TILs), and notably memory cells and cytotoxic T lymphocytes (CTLs) [4].

Otherwise, CRC is considered to be a cold tumor with a low number of neoantigens and an immune-excluded or immune-desert TME with absent or inactive TILs [5]. Therefore, while immune checkpoint inhibitors—such as anti-PD-1, anti-PD-L1, and anti-CTLA-4 agents—have demonstrated efficacy as frontline therapy as reported in the KEYNOTE-177 study in patients with previously-treated dMMR CRC who have progressed on fluoropyrimidines, oxaliplatin, and irinotecan [6], they fail to provide benefit to 95% of patients with mCRC defined as proficient mismatch repair (pMMR) or microsatellite stable (MSS) tumors.

Five single-arm trials (phase Ib KEYNOTE 012 and 028, and phase II KEYNOTE 016, 164, and 158) investigated the anti-PD-1 agent pembrolizumab across different tumor types, including 149 patients with previously-treated advanced CRC, 59 of whom presented MSS/pMMR tumors. Forty-one patients with metastatic MSI-H or MSS carcinoma included in the phase II KEYNOTE-016 clinical trial received treatment with pembrolizumab [7].

The overall response rate (ORR) in patients with dMMR was 71% (5 of 7 patients) and 40% (4 of 10 patients) for non-CRC and CRC, respectively, while the ORR in patients with pMMR was 0% (0 of 18 patients). The immune-related progression-free survival (PFS) rate at 20 weeks was 67% (4 of 6 patients), 78% (7 of 9 patients), and 11% (2 of 18 patients), for dMMR non-CRC, dMMR CRC, and pMMR CRC, respectively. These data support the hypothesis that, regardless of the primary tumor origin, the large proportion of mutant neoantigens in dMMR tumors make them sensitive to immune checkpoint inhibitors. [8]. However, it should be noted that, similarly to dMMR/MSI tumors, response to pembrolizumab has been reported in a patient with MSS mCRC harboring *POLE* mutations (which encodes the DNA polymerase ε responsible for lead strand DNA replication) [9]. This antitumor activity might be mediated by an increase in the expression of cytotoxic T-cell markers and effector cytokines and a higher intratumoral CD8^+^ lymphocyte infiltration [10].

Thus, given that most patients with MSS mCRC do not respond to immunotherapy or do not have durable clinical responses, it is necessary to obtain predictors of response to immunotherapy and rational immunotherapy-based combination therapies. The cornerstone of developing biologically sound combination therapies for MSS CRC is to unravel the mechanisms underlying primary or acquired resistance to immunotherapy and enhance efficacy. Novel strategies involve prompting the immune cycle at various levels, either by heightening the TMB and the number of neoantigens, by impacting the interferon-γ (IFN-γ) signature with inhibition of immunosuppressive ligands expression, or by transforming the TME into an immune response phenotype with effective immune cells.

In this review, we present the efforts to date to develop immunotherapy-based combinations for MSS mCRC in the clinical setting. A retrospective review of the literature was conducted by searching PubMed, MEDLINE, Cochrane, and ClinicalTrials.gov databases, using the following MeSH terms: “colorectal cancer”, “immunotherapy”, and “pMMR” or “proficient mismatch repair”.

## 2. Immunotherapy-Based Combinations in pMMR/MSS mCCR

**Figure 1:**
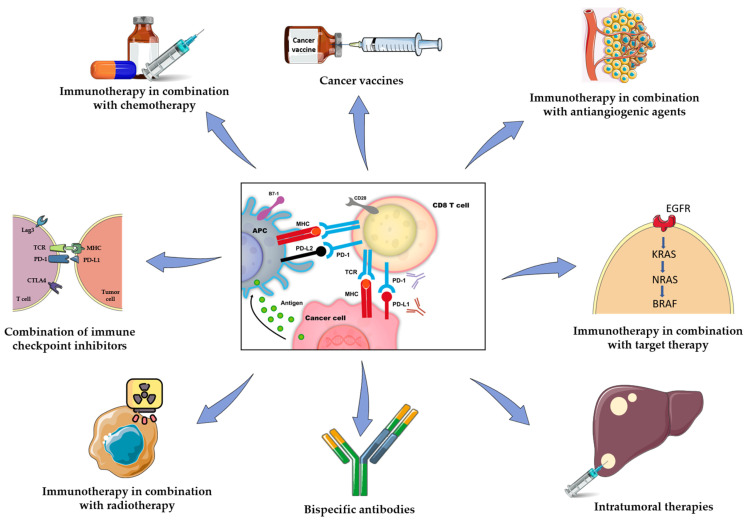
Approaches investigated so far to overcome immune resistance and develop an effective immune response against tumor cells for MSS mCRC. Created with smart.servier.com, accessed on 8 December 2021.

Novel immunotherapy-based approaches have been developed under the rationale of overcoming immune resistance and developing an effective immune response against tumor cells, such as combined strategies of immune checkpoint inhibition, immunotherapy-based combinations with chemotherapy and targeted therapy, radiation therapy, vaccines, and intratumoral strategies such as oncolytic viruses and bispecific antibodies (Figure 1, Table 1).

Inhibition of the PD-1/PDL-1 axis alone has proven insufficient for pMMR/MSS mCRC. Novel and a range of immunotherapy-based approaches have been developed based on the rationale of overcoming immune resistance and developing an effective immune response against tumor cells.

### 2.1. Combination of Immune Checkpoint Inhibitors

Given that the inhibition of the PD-1/PD-L1 axis alone has proven insufficient for pMMR/MSS mCRC, combined blockade with immunotherapy strategies has been explored to overcome immune resistance in this setting.

In the MSS refractory setting, combined immune checkpoint inhibition with the anti-PDL-1 agent durvalumab plus the anti-CTLA-4 agent tremelimumab showed modest results in terms of response rate (0.8%) and a trend in overall survival (OS) benefit with a 2.5-month improvement for the combination over best supportive care [11]. Exploratory analyses showed that patients presenting plasma TMB of 28 variants per megabase or more (21% of MSS tumors) had the greatest OS benefit (hazard ratio [HR]: 0.34, *p* = 0.004). Results from the phase I first-in-human clinical trial testing the anti-LAG-3 antiboy favezelimab and the anti-PD-1 pembrolizumab in previously treated patients with advanced MSS CRC were recently reported [12]. Of 89 patients receiving the combined blockade, four patients presented partial response and one patient achieved complete response. Median duration of response was 10.6 months (range 5.6–12.7) and toxicity was manageable.

### 2.2. Immunotherapy in Combination with Chemotherapy

#### 2.2.1. Immunotherapy in Combination with Chemotherapy and Anti-VEGF Agents

Strategies based on the combination of chemotherapy with immunotherapy to overcome immune resistance of pMMR/MSS CRC have been widely explored, given that some chemotherapy agents may have an immune-stimulatory effect. There is evidence supporting that 5-fluorouracil (5-FU), the backbone of chemotherapy in CRC, induces apoptosis of myeloid-derived suppressor cells (MDSCs) and therefore favors tumor infiltration by CTLs [30,31]. Moreover, oxaliplatin induces tumor cell death, rendering tumor cells and cancer-specific antigens recognizable for the immune system and immunogenic cell death in cell lines derived from colorectal tumors by translocating the chaperone molecule calreticulin from the lumen of the endoplasmic reticulum to the cell surface [32,33,34,35]. An in vivo assay using an immune checkpoint blockade-resistant mouse xenograft model of colon cancer showed antitumor response when oxaliplatin was combined with immune checkpoint inhibitors [36].

Bevacizumab, an antiangiogenic agent widely used in the frontline treatment in combination with chemotherapy, inhibits the VEGF/VEGFR pathway upon binding to vascular endothelial growth factor A (VEGF-A). VEGF/VEGFR blockade leads to vasculature normalization, permitting increased tumor infiltration of T cells, and activation of effector immune cells through stimulation of the maturation of dendritic cells (DCs) and reduction of the expansion of Tregs and MDSCs [37,38,39,40,41].

These data provide the rationale to test the combination of chemotherapy with an antiangiogenic agent and immunotherapy in the clinical setting. However, the MODUL trial, which evaluated maintenance treatment with 5-FU and atezolizumab after first-line induction therapy with FOLFOX in patients with metastatic *BRAF* wild-type CRC did not show a significant difference in either PFS or OS [13]. More recently, results from the AtezoTRIBE study, in which frontline FOLFOXIRI and bevacizumab in combination with atezolizumab was compared with chemotherapy and bevacizumab alone, have been reported [14]. Of 218 patients included, 199 patients presented a diagnosis of advanced MSS CRC. In this subgroup, PFS was 12.9 months for the group treated with the combination and 11.4 months for the control group (*p* = 0.07). The combination of chemotherapy and immunotherapy in the frontline setting has also been evaluated in the phase II/III CA2099X8 trial evaluating FOLFOX combined with bevacizumab and nivolumab versus FOLFOX and bevacizumab (NCT03414983) and in the phase Ib/II COLUMBIA-1 trial comparing FOLFOX and bevacizumab in combination with durvalumab and oleclumab versus FOLFOX and bevacizumab (NCT04068610), for which outcomes are yet to be reported. Although the POCHI trial (NCT04262687) is currently exploring the combination of CAPOX and bevacizumab with pembrolizumab as first-line therapy in eligible patients with MSS mCRC who present a high immune infiltrate, no biomarker-based selection has been defined so far to optimize patient identification for this combined strategy.

In the chemo-refractory setting, the BACCI trial evaluated the addition of atezolizumab to capecitabine and bevacizumab [15]. Of 82 patients included in the experimental group and 46 patients in the control group, 86% and 87% presented MSS status. There were no statistical differences for ORR or PFS in the MSS sub-group between these treatments.

#### 2.2.2. Immunotherapy in Combination with Chemotherapy and Anti-EGFR Agents

Standard therapy for the treatment of metastatic RAS/BRAF wild-type CRC includes the use of agents targeting the epidermal growth factor receptor (EGFR) in combination with chemotherapy.

Cetuximab, a chimeric IgG1 antibody, induces antibody-dependent cellular toxicity (ADCC) triggered by Fcγ receptors on immune cells and promotes expression of MHC class II molecules on dendritic cells (DCs) [42,43,44], and is approved as part of management of CRC in several settings. The addition of avelumab to FOLFOX and cetuximab regardless of the microsatellite status in the frontline setting has been explored in the phase II AVETUX clinical trial. Although the combined treatment presented a response rate of 79.5%, the actuarial PFS at 12 months was 40% and did not reach its primary endpoint (increase in progression-free rate at 12-month from 40% to 57%) [16]. The combination of an intensified regimen using the triple-drug chemotherapy regimen FOLFOXIRI with either the anti-EGFR cetuximab or panitumumab has been investigated in the MACBETH and VOLFI trials, respectively, showing a higher ORR. The addition of the anti-PD-L1 avelumab to FOLFOXIRI and cetuximab in the frontline setting for MSS RAS/BRAF wild-type mCRC is being evaluated in the phase II AVETRIC trial (NCT04513951). After induction therapy, maintenance treatment with 5-FU plus cetuximab and avelumab is scheduled until disease progression.

The use of immunotherapy for patients with *RAS/BRAF* wild-type MSS CRC is being investigated beyond frontline therapy in a phase II clinical trial evaluating the combination of nivolumab and ipilimumab with panitumumab in patients who have received one or two prior lines of systemic treatment (NCT03442569). Based on the activity reported following rechallenge with EGFR-containing regimens for patients who previously experienced benefit with anti-EGFR agents [45], the phase II single-arm CAVE-Colon trial addresses the potential benefit of combining avelumab with cetuximab in the third-line setting, with no selection for microsatellite status [46]. Median OS of 11.6 months and a median PFS of 3.6 months were achieved. Plasma circulating tumor DNA (ctDNA) analysis before treatment may allow selection of patients who could benefit, as median OS and PFS were longer in patients with *RAS/BRAF* WT ctDNA (17.3 vs. 10.4 months, *p* = 0.02, and 4.1 vs. 3.0 months, *p* = 0.004, respectively). Of 77 patients included, 71 (92%) presented advanced MSS CRC. These preliminary results require further exploration in a randomized phase III setting in selected patients.

#### 2.2.3. Immunotherapy in Combination with Temozolomide

Temozolomide (TMZ) is an oral alkylating agent whose mechanism of action lies in the methylation of DNA strands at the O6 position of guanine. This methylation damages DNA, provoking inhibition of replication and apoptosis. The O6-methylguanine methyltransferase (MGMT) enzyme coded by the MGMT gene reduces the therapeutic efficacy of TMZ [47]. Therefore, the effectiveness of TMZ in patients with advanced CRC is restricted to those tumors with MSS status, epigenetic silencing of MGMT mediated by the methylation of its promoter region, and no or low expression [48,49]. In a phase II clinical trial evaluating temozolomide in heavily pretreated patients whose tumors harbor MGMT promoter methylation, modest activity was observed, with an ORR of 10% and a disease control rate (DCR) of 32% [50]. Acquired resistance to TMZ might be mediated by the emergence of mutations in the MMR machinery and the induction of hypermutation [51,52,53].

TMZ could therefore be useful as priming agent for immune-sensitization in pMMR/MSS CRC. Results were recently reported for the phase II MAYA trial (NCT03832621), in which patients with chemo-refractory MSS CRC presenting *MGMT* silencing and benefiting from two cycles of TMZ, were treated with nivolumab and ipilimumab in combination with TMZ [17]. Actuarial PFS at 8 months was 36%, ORR was 42% and median OS was 18.4 months, while paired biopsies showed acquired TMB-high after treatment initiation. The ARETHUSA trial (NCT03519412) in which patients with MSS CRC, who are MGMT-negative and promoter methylation-positive by immunohistochemistry and who present a TMB > 20 mutations per megabase after treatment with TMZ, will be treated with pembrolizumab, is currently recruiting.

### 2.3. Immunotherapy in Combination with Antiangiogenic Agents

Beyond the combination of chemotherapy and antiangiogenic agents with immunotherapy, combination of an antiangiogenic agent with immunotherapy but without chemotherapy has also been explored.

The potential immune-modulating effect of lenvatinib in combination with PD-1/L1 signal inhibitors has been studied in preclinical models. Combination of pembrolizumab and lenvatinib in syngeneic murine models led to activation of CD8^+^ T cells through reduction of TAMs, which are immune regulators in the TME, and to activation of the interferon pathway that resulted in a synergistic effect with greater reduction in tumor volume and higher response rate [54,55]. A non-randomized phase II trial conducted in patients with previously treated MSS CRC receiving pembrolizumab and lenvatinib showed promising antitumor activity with an ORR of 22%, a median PFS of 2.3 months, and a manageable safety profile [18]. The randomized phase III trial comparing this immunotherapy-based combination versus standard of care (TAS102 or regorafenib) is currently recruiting (NCT04776148).

The combination of the multi-kinase inhibitor regorafenib with immunotherapy has also been explored, based on the ability of regorafenib to generate a more inflamed TME due to reduction of vessel permeability and TME infiltration by TAMs [56], the reduction of TAM proliferation and reprogramming into a subpopulation of cells with antitumor effects [57], and the blockade of IFN-γ-induced PD-L1 and IDO1 expression while maintaining MHC-I expression [58].

In light of these results, a phase Ib study evaluated the safety of nivolumab and regorafenib in 25 patients with pMMR CCR, resulting in a low ORR of 4.8%, a median PFS of 4.3 months (95% CI 2.1–15.6), and a median OS of 11 months [19]. The REGOMUNE trial, a single-arm, phase II study examined the efficacy and safety of regorafenib 160 mg in combination with anti-PD-L1 blockade with avelumab in 48 patients with pMMR CRC. Although there were no responses, 23 patients presented stable disease (54%), with a median PFS of 3.6 months (95% CI 1.8–5.4), and a median OS of 10.8 months (95% CI 5.9–NA). Increased tumor infiltration by CD8^+^ T cells at cycle 2 was associated with better outcomes [20].

### 2.4. Immunotherapy in Combination with Radiotherapy

Radiotherapy can enhance the immune response via multiple mechanisms. Locally, by damaging DNA, radiotherapy induces tumor-cell death—promoting antigen presentation and tumor-associated antigen-MHC complexes, T-cell recruitment and activation, and upregulation of inflammatory cytokines—enhancing the susceptibility of irradiated tumor cells to the immune system. Distantly, radiotherapy can induce an abscopal effect, which results in radiological responses outside the irradiated field mediated by an increase of immune-cell tumor infiltrates and direct presentation of tumor antigens [59].

There is preclinical evidence indicating that the pro-immune effects of locoregional radiotherapy can be synergistically augmented with immunostimulatory monoclonal antibodies to boost a regression in both irradiated tumor lesions and off-target lesions. This abscopal effect provides a basis for the rational design of combination therapy [60,61].

Double immune checkpoint inhibition and radiotherapy with the aim of obtaining an abscopal response was tested in a single arm phase II trial including evaluation of non-irradiated lesions. The combination of durvalumab and tremelimumab and radiotherapy resulted in an ORR of 8.3%, a median PFS of 1.8 months, and a median OS of 11.4 months, not meeting the prespecified endpoint criteria. Nonetheless, combined radiotherapy with durvalumab and tremelimumab led to shrinkage of distant, nonirradiated tumors in MSS mCRC, with manageable safety. Plus, a heightened CD8^+^ T-cell activation, differentiation and proliferation was noted in patients with objective response [21]. In another phase II trial evaluating radiotherapy with ipilimumab and nivolumab, the combined treatment led to shrinkage of nonirradiated distant tumors with an ORR of 12.5% [22].

Other phase II clinical trials are currently assessing the activity of immune checkpoint inhibitors in combination with different strategies of locoregional radiation (standard radiotherapy stereotactic body radiotherapy and radiofrequency ablation) in the same setting (NCT02992912, NCT02437071, NCT03122509). Interestingly, the dose and fractionation employed may have an impact in the ability of radiotherapy to synergize with immunotherapy, since immune response genes have been shown to be expressed differentially in irradiated tumors according to the regimen used [62], while the ideal timing of administration with radiation may be dependent on the mechanism of action of the immunotherapy used [63].

### 2.5. Immunotherapy in Combination with Target Therapy

#### 2.5.1. Immunotherapy in Combination with MAPK Signaling Inhibitors

Overexpression and activation of the RAS/BRAF/MEK/ERK pathway are commonly detected in CRC [64,65]. There is growing evidence that dysregulation of the MAPK pathway is associated with an immunosuppressive phenotype. Previous investigations have reported that MAPK signaling is essential for T-cell development, activation, proliferation, and survival and that MAPK signaling may control PD-L1 and CTLA-4 expression [66,67,68,69,70,71]. This provides the biological rationale to explore the synergic effect of immunotherapy agents and selective inhibitors of the RAS/BRAF/MEK/ERK pathway in pMMR/MSS mCRC.

##### Immunotherapy in Combination with KRAS Inhibitors

Analyses of the composition and functional distribution of the immune, fibroblastic, and angiogenic microenvironment of 1338 colorectal tumors from three independent cohorts unveiled that the CMS3 subtype, termed metabolic and that are enriched in tumors with *KRAS* mutations, displayed low immune and inflammatory signatures [72].

Despite extensive efforts at targeting KRAS, to date only *KRAS* G12C mutations have been demonstrated to be targetable, with a promising ORR of 32%, a DCR of 88% and median PFS of 6.3 months were observed in pretreated patients with tumors harboring *KRAS* G12C mutations who received the KRAS G12C inhibitor sotorasib [73]. In the metastatic CRC sub-cohort, ORR, DCR, and PFS were 7%, 74%, and 4.0 months, respectively. Preclinical data using immune-competent mice showed that treatment with sotorasib induced a pro-inflammatory TME through increased interferon signaling, chemokine production, antigen processing, cytotoxic and natural killer cell activity, and stimulation of innate immune system [74]. Plus, combined treatment with immune-checkpoint inhibitors resulted in durable responses. Characterization of the molecular mechanisms underlying the synergistic effect of the combined blockade through immunophenotype analyses revealed an increased infiltration of CD8^+^ T cells, an increased number of total and proliferating CD3^+^ T cells, and the establishment of memory T cell response. Based on these in vivo experiments, combined inhibition of *KRAS* G12C and the PD-1/PD-L1 axis is being tested and clinical trials are currently recruiting (NCT03600883 and NCT04699188).

Efficacy of the combined treatment of KRAS and PD-1/PD-L1 inhibition with the SHP2 inhibitor TNO155 is also being tested in the phase Ib/II trial (NCT04699188), as SHP2 phosphatase is known to suppress T cell activation through CD28 dephosphorylation upon PD-1/PD-L1 ligation [75]. Therefore, triplet combination may exert increased antitumor activity as it may decrease intracellular PD-1 signaling and lead to a less suppressive TME.

##### Immunotherapy in Combination with BRAF Inhibitors

*BRAF* mutations in CRC occur in up to 15% of patients. The most frequent mutation is V600E, in which a valine in codon 600 is substituted for a glutamate at the amino acid level, and results in sustained *RAS/RAF/MEK/ERK* pathway signaling through constitutive activation of *BRAF* kinase [76,77]. Patients with mCRC harboring *BRAF* mutations present a poor prognosis and dismal median survival [78].

The single inhibition of BRAF in mCRC has proved ineffective due to feedback reactivation of EGFR and of the downstream immune-suppressive MAPK cascade [79,80]. However, the co-administration of the BRAF inhibitor with the EGFR inhibitor cetuximab, tested in the BEACON trial, resulted in significantly longer overall survival and a higher response rate than standard therapy, with a safety toxicity profile establishing a new standard of care for patients with previously treated *BRAF*-V600E-mutant CRC [81].

Paired tumor biopsies in patients with CRC harboring *BRAF*-V600E mutations and treated with triple-pronged therapy targeting BRAF, EGFR, and MEK showed that an increase in T-cell infiltration occurred after blockade of the MAPK pathway via BRAF inhibition [82]. Drawing from these findings, in vivo experiments using immune-competent murine models were performed. In vivo assays demonstrated the efficacy of the combined blockade of BRAF and the PD-1/PD-L1 axis over these strategies separately [82]. According to this rationale, the potential cooperativity between BRAF-targeting and the immune response is being further explored in two phase I trials testing the combination of the BRAF inhibitor dabrafenib, the MEK inhibitor trametinib and the anti-PD-1 antibody spartalizumab (NCT03668431), as well as the combination of dabrafenib, the ERK inhibitor LTT462, and spartalizumab (NCT04294160).

##### Immunotherapy in Combination with MEK Inhibitors

MEK inhibition in preclinical models led to upregulation of MHC I and a higher number of effector-phenotype antigen-specific CD8^+^ T cells within the tumor. In vivo studies combining MEK inhibition with blockade of the PD-1/PD-L1 axis resulted in synergistic and durable tumor regression in murine models of *KRAS*-mutated CRC [83,84].

Clinical exploration of this strategy in a phase Ib clinical trial evaluating the combination of atezolizumab plus the MEK inhibitor cobimetinib in 84 patients with chemorefractory CRC resulted in an ORR of 8% [23]. Interestingly, of seven patients presenting partial response, six had MSS tumors. Based on these results, the IMBLAZE370 phase III trial (COTEZO) randomized 363 patients with chemorefractory CRC to receive either atezolizumab plus cobimetinib, atezolizumab in monotherapy or regorafenib. However, the trial failed to meet its primary endpoint. Median OS was 8.9 months for the experimental arm, 8.5 months for the control arm with regorafenib, and 7.1 months for patients receiving atezolizumab. Moreover, there were no differences in terms of PFS and ORR across the treatment arms [24].

#### 2.5.2. Immunotherapy in Combination with Inhibition of the PI3K/AKT/mTOR Pathway

PI3K pathway activation is often present in CRC, derived from the loss of *PTEN* and *PIK3CA* mutations and is associated with immune checkpoint upregulation [85]. Activating mutations in *PIK3CA* in in vivo assays confers resistance to anti-PD-1 agents, while coadministration with a PI3K inhibitor overcame resistance [86]. Meanwhile, PI3Kγ and PI3Kδ isoforms might reprogram TAMs towards an immune-tolerant phenotype and PI3K inhibition would subsequently relieve Treg-mediated immunosuppression [87,88]. As a result of these findings, a phase I/II clinical trial exploring the potential efficacy of the combination of nivolumab with the PI3K inhibitor copanlisib is actively recruiting [25].

### 2.6. Bispecific Antibodies

Bispecific antibodies designed to recognize two different epitopes or antigens aim to address the host immune activity towards tumor cells by binding both tumor-enriched antigens (i.e., CEA, CEACAM, EpCAM, HER2, or CD276 antigen) and immune cells (mainly T cells via the CD3 receptor) and are being explored under this rationale in different types of solid tumors [89]. Some of these drugs are also being tested in combination with other agents, such as immune checkpoint inhibitors, to enhance antitumor activity [90].

Cibisatamab (RO6958688), a novel T-cell bispecific antibody targeting CEA on colorectal tumor cells and CD3 on T cells, is the bispecific antibody that is currently the furthest along the clinical development path for MSS mCRC. In preclinical models, cibisatamab displayed potent antitumor activity, leading to increased intratumoral T cell infiltration and activation and PD-1/PD-L1 upregulation [91]. Furthermore, blockade of the PD-1/PD-L1 axis in in vitro assays maximized tumoral T cell killing, directed by an anti-CEA bispecific antibody [92].

In two ongoing dose-escalation phase I studies, cibisatamab was given as monotherapy or in combination with atezolizumab in patients with solid tumors expressing CEA [26]. Consistent with its known mechanism of action, tumor inflammation was observed in CT scans on days of administration of the first dose of cibisatamab. Of 68 patients with mCRC treated with cibisatamab (0.05 to 600 mg) and of 38 patients with mCRC treated with cibisatamab (5–160 mg) in combination with atezolizumab, 10 (28%) and 6 (60%) respectively presented a metabolic partial response by PET scan four to six weeks after treatment initiation. Confirmed radiologic ORR for each group was 6% and 12%, respectively. The most notable toxicities associated with cibisatamab are tumor inflammatory events and cytokine release syndrome due to immune-cell activation, occurring more often upon first exposure to cibisatamab.

Other bispecific T-cell engager antibodies are under development for MSS mCRC, including RO7122290 (a bispecific antibody-like fusion protein which connects a trimeric split 4-1BBL domain to a one-armed human IgG1 antibody directed against fibroblast activation protein) in combination with cibisatamab (NCT04826003) and BLYG8824A (a bispecific humanized IgG1 antibody binding the extracellular domain of LY6G6D and the CD3 subunit of the T cell-receptor) (NCT04468607).

### 2.7. Cancer Vaccines

Cancer vaccines are designed to trigger an intense response of the immune system to tumor-specific antigens. DC vaccines can be obtained by isolating DCs from the host, pulsing them ex vivo with tumor-associated antigens (TAAs) derived from autologous tumor lysate and then reinfusing them into the patient after activation. Upon infusion, the DCs present the TAAs on the cell surface, causing the activation of T cells and resulting in a cancer-specific immune response.

Despite the underlying rationale, disappointingly, little or no clinical efficacy has been observed so far with this strategy in MSS mCRC. Two phase II clinical trials, using autologous tumor lysate DC vs. best supportive care in patients with pretreated CRC [27] and a peptide vaccine based on the combination of five peptides derived from tumor-associated antigens (RNF43, TOMM34, KOC1, VEGFR1, and VEGFR2) with oxaliplatin-based chemotherapy in the frontline setting [28]; respectively, demonstrated the generation of a tumor-specific immune response but failed to demonstrate clinical benefit. Finally, the combination of vaccines with checkpoint inhibitors will probably overcome the immunosuppressive TME through enhancement of the immune response, due to intensified immunogenicity and an increased number of tumor-specific T cells. Preclinical assays showed that dual blockade of PD-1 and CTLA-4 combined with GM-CSF gene-transfected tumor cell vaccine (GVAX) in murine models of CRC resulted in tumor rejection in 100% of mice [93].

### 2.8. Intratumoral Therapies

Intratumoral administration of selected drugs in combination with other immune strategies may enhance antitumor response through attraction of immune cells to the tumor. Stimulator of interferon genes (STING) acts as an innate immune sensor for cytosolic DNA and its activation is crucial to trigger the type I interferon response and the antitumoral immune response by CD8^+^ T cells [94]. Intratumoral injection of synthetic STING agonists in murine CRC models resulted in impaired tumor growth mediated by an increase in intratumoral CD8^+^ T cell infiltration [95]. This preclinical therapeutic effect is now being explored in phase I clinical trials (NCT03843359).

Oncolytic viruses are genetically modified to ensure they are unable to damage the host’s normal tissues, which may act as double-edged swords against tumors through direct cytolysis of cancer cells and the induction of antitumor immunity. A phase I/II clinical trial evaluated the safety and the antitumor activity of the genetically engineered oncolytic herpes simplex virus NV1020 administered via weekly hepatic artery infusion, followed by two or more cycles of conventional chemotherapy, in pretreated chemorefractory CRC patients with liver metastases [29]. Among the 22 patients treated, 50% showed stable disease after NV1020 administration and 68% presented DCR after treatment with chemotherapy (1 partial response and 14 stable disease); median time to progression was 6.4 months and median OS was 11.8 months. Talimogene laherparepvec—a modified herpes simplex virus approved for intratumoral treatment of unresectable recurrent cutaneous, subcutaneous, and nodal melanoma—is being tested in combination with atezolizumab for patients with CRC with liver involvement in a phase Ib trial (NCT03256344).

Other intratumoral strategies—including but not restricted to Toll-like receptor agonists, immune receptor agonists, immune cells, cytokines, and mRNA encoding cytokines—are also being explored.

## 3. Conclusions

Despite the clinical benefit of immunotherapy observed in patients with MSI mCRC, to date neither immunotherapy agents as monotherapy nor immunotherapy-based combinations have been approved for MSS mCRC, and a long road lies ahead for this entity.

Immunotherapy-based strategies that are currently under development include, among others, combination with chemotherapy, radiotherapy, or targeted therapies; bispecific antibodies; cancer vaccines; and intratumoral therapies. These strategies aim at turning an effective immune response against tumor cells. Unravelling the pathways mediating immune resistance in MSS colorectal tumors remains an ongoing challenge, along with optimizing rational immunotherapy-based combinations to render them amenable for these strategies, ameliorating patient selection, and improving prevention and management of immune-related adverse events such that the implementation of immunotherapy in this setting moves forward successfully.

Preclinical functional evidence obtained through the use of patient-derived models—including patient-derived organoids and patient-derived xenografts—and the use of reverse translational studies may illuminate the path forward.

## Figures and Tables

**Table 1:** Summary of clinical trials investigating the use of immunotherapy-based combinations for MSS mCCR.

Study	Treatment	Phase	Setting	Sample Size	ORR	Median PFS	Median OS
**Combination of immune checkpoint inhibitors**							
CCTG CO.26 [11]	Durvalumab + Tremelimumab vs. BSC	II	Refractory	119 vs. 61	0.5% vs. 0%	1.8 vs. 1.9 months	6.6 vs. 4.1 months
NCT02720068 [12]	Pembrolizumab + Favezelimab	I	Refractory	80	6.3%	2.1 months	8.3 months
**Immunotherapy in combination with chemotherapy**							
***Immunotherapy in combination with chemotherapy and anti-VEGF agents***							
MODUL [13]	FOLFOX + Bevacizumab followed by 5-FU + Bevacizumab + Atezolizumab vs. 5-FU + Bevacizumab	II	First line	297 vs. 148	NA	7.2 vs. 7.39 months	22 vs. 21.9 months
ATEZOTRIBE [14]	FOLFOX + Bevacizumab + Atezolizumab vs. FOLFOX + Atezolizumab	III	First line	132 vs. 67	59% vs. 64%	12.9 vs. 11.4 months	NA
CA2099 × 8	FOLFOX + Bevacizumab + Nivolumab vs. FOLFOX + Bevacizumab	II/III	First line	Active	NA	NA	NA
COLUMBIA-1	FOLFOX + Bevacizumab + Durvalumab + Oleclumab vs. FOLFOX + Bevacizumab	II	First line	Active	NA	NA	NA
NIVACOR (NCT04072198)	FOLFOXIRI + Bevacizumab + Nivolumab	II	First line	Recruiting	NA	NA	NA
BACCI (NCT0287319) [15]	Capecitabine + Bevacizumab + Atezolizumab vs. Capecitabine + Bevacizumab	II	Refractory	82 vs. 46	8.5% vs. 4.3%	4.4 vs. 3.3 months	NA
***Immunotherapy in combination with chemotherapy and anti-EGFR agents***							
AVETRIC (NCT04513951)	FOLFOXIRI + Cetuximab + Avelumab	II	First line	Recruiting	NA	NA	NA
AVETUX (NCT03174405) [16]	FOLFOX + Cetuximab + Avelumab	II	First line	43	79.5%	11.5 months	NA
**Immunotherapy in combination with temozolomide**							
ARETHUSA (NCT03519412)	Temozolomide followed by Pembrolizumab	II	Refractory	Recruiting	NA	NA	NA
MAYA [17]	Temozolomide + Nivolumab + Ipilimumab	II	Refractory	33	42%	7.1 months	18.4 months
**Immunotherapy in combination with antiangiogenic agents**							
LEAP-005 [18]	Pembrolizumab + Lenvatinib	II	Refractory	32	22%	2.3 months	7.5 months
REGONIVO [19]	Nivolumab + Regorafenib	Ib	Refractory	25	36%	7.9 months	NA
REGOMUNE [20]	Avelumab + Regorafenib	II	≥2 lines	48	0%	3.6 months	10.8 months
**Immunotherapy in combination with radiotherapy**							
NCT03122509 [21]	Radiotherapy + Durvalumab + Tremelimumab	II	>2 lines	24	8.3%	1.8 months	11.4 months
NCT03104439 [22]	SBRT + Nivolumab + Ipilimumab	II	>2 lines	40	12,5%	NA	NA
NCT02992912	SABR + Atezolizumab	II	Refractory	Recruiting	NA	NA	NA
**Immunotherapy in combination with MAPK signaling inhibitors**							
***Immunotherapy in combination with KRAS inhibitors***							
CodeBreaK 100	AMG 510 +/− AntiPD-1/L1	I/II	Refractory	Recruiting	NA	NA	NA
NCT04699188	JDQ443 +/− TNO155 +/- Spartalizumab	I/II	Refractory	Recruiting	NA	NA	NA
***Immunotherapy in combination with BRAF inhibitors***							
NCT03668431	Dabrafenib + Trametinib (MEK) + Spartalizumab	II	Refractory	Recruiting	NA	NA	NA
NCT04294160	Dabrafenib + LTT462 (ERK) + Spartalizumab	I	Refractory	Recruiting	NA	NA	NA
***Immunotherapy in combination with MEK inhibitors***							
NCT01988896 [23]	Cobimetinib + Atezolizumab	I/Ib	Refractory	84	8%	1.9 months	9.8 months
IMBlaze 370 [24]	Atezolizumab + Cobimetinib vs. Atezolizumab vs. Regorafenib	III	Refractory	183 vs. 90 vs. 90	3%	1.9 vs. 1.9 vs. 2 months	8.9 vs. 7.1 vs. 8.5 months
**Immunotherapy in combination with inhibition of the PI3K/AKT/mTOR pathway**							
NCT03711058 [25]	Copanlisib + Nivolumab	I/II	Refractory	Recruiting	NA	NA	NA
**Biespecific antibodies**							
NCT02324257 [26]	Cibisatamab	I	Refractory	68	6%	NA	NA
NCT02650713 [26]	Cibisatamab + Atezolizumab	I	Refractory	38	12%	NA	NA
NCT04826003	RO7122290 + Cibisatamab + Obinutuzumab	I	Refractory	Recruiting	NA	NA	NA
NCT04468607	BLYG8824A	I	Refractory	Recruiting	NA	NA	NA
**Vaccines**							
NCT01413295 [27]	Dendritic cell vaccine vs. BSC	II	>2 lines	28 vs. 24	0% vs. 0%	2.7 vs. 2.3 months	6.2 vs. 4.7 months
FXV [28]	HLA-A*2402-restricted peptides + oxaliplatin-based chemotherapy	II	First line	50 (HLA-A*2402-matched)	62%	7.2 months	20.7 months
		II	First line	46 (HLA-A*2402-unmatched)	60.9%	8.7 months	24.0 months
**Intratumoral therapies**							
NCT00149396 [29]	Hepatic artery infusion of NV1020 followed by conventional chemotherapy	I/II	Refractory	22	4.5%	6.4 months	11.8 months
NCT03256344	Talimogene laherparepvec + Atezolizumab	Ib	Refractory	Active	NA	NA	NA

Abbreviations: BSC, best supportive care; MSS, microsatellite stable; NA, not announced; ORR, overall response rate; OS, overall survival; PFS, progression-free survival; RT, radiotherapy; SBRT/SABRT, stereotactic ablative radiotherapy.
